# Toward equality: Including non-human animals in studies of lived religion and nonreligion

**DOI:** 10.1177/00377686231170993

**Published:** 2023-05-25

**Authors:** Lori G BEAMAN, Lauren STRUMOS

**Affiliations:** University of Ottawa, Canada; Leipzig University, Germany; University of Ottawa, Canada

**Keywords:** anthropocentrism, climate crisis, human–animal relations, nonreligion, sociological theory, anthropocentrisme, crise climatique, non-religion, relations homme-animal, théorie sociologique

## Abstract

This article explores how sociologists of religion can respond to ‘the animal turn’ in studies of lived religion and nonreligion. We begin by considering how sociology has neglected the place of non-human animals and the ‘more than human’ in social life. We then turn to the sociology of religion, where animals have often been devalued or ignored as irrelevant to understanding religion in society. We argue that it is necessary to consider the ways in which human activities are shaped by non-human animals. This does not mean that animals should be thought of as nonreligious or religious. We contend that the failure to incorporate non-human animals in sociological considerations of religion and nonreligion replicates a hierarchical model, which sees human life as above or higher than non-human life and calls our attention to the place of sociological research amid the climate crisis.

## Résumé

Cet article explore comment les sociologues de la religion peuvent répondre au « tournant animal » dans les études sur la religion et la non-religion vécues. Nous commençons par examiner comment la sociologie a négligé la place des animaux non humains et du « plus qu’humain » dans la vie sociale. Nous nous tournons ensuite vers la sociologie de la religion, où les animaux ont souvent été dévalorisés ou ignorés comme n’étant pas pertinents pour comprendre la religion dans la société. Nous soutenons qu’il est nécessaire de considérer les façons dont les activités humaines sont façonnées par les animaux non humains. Cela ne signifie pas que les animaux doivent être considérés comme non religieux ou religieux. Nous soutenons que l’échec de l’incorporation des animaux non humains dans les considérations sociologiques de la religion et de la non-religion reproduit un modèle hiérarchique, qui voit la vie humaine au-dessus ou plus haut que la vie non humaine et attire notre attention sur la place de la recherche sociologique dans la crise climatique.

## Introduction

This article explores how sociologists of religion can respond to ‘the animal turn’ in studies of religion and nonreligion. It starts by considering how sociology more broadly has neglected the place of animals in social life. We then turn to the sociology of religion, where animals have often been devalued or ignored as irrelevant to understanding religion in society. In response, this article proposes to examine the ways in which human activities are shaped by non-human animals. At a broader level, we think it is important to link the failure to integrate non-human/human–animal relations with hierarchical understandings of life on earth and the climate crisis we currently face. Ultimately, without a reconfiguration of our understanding of society to both acknowledge and correct the exclusionary impact of this hierarchy, sociology and sociology of religion are ill equipped to respond to the climate crisis.

The recent ‘animal turn’ in the social sciences is an opportunity for sociologists of religion to critically re-engage with their object of study. This re-engagement entails serious consideration of the place of non-human animals in social life, and a reflexive understanding of animals’ absence in sociology from the time of the field’s founding. Altglas and Wood state that a critical sociology of religion ‘entails thinking about the conditions in which we produce sociological knowledge and how this knowledge is affected by these conditions; otherwise, we may simply produce a knowledge serving the interests of certain social groups and institutions’ (2018: 28). In this article, we address the anthropocentric conditions that produced a sociology of religion devoid of non-human animals.^1^ Furthermore, we contend that the failure of sociologists to incorporate animals in considerations of religion and nonreligion demonstrates – and perpetuates – the human/nature dualism of Western thought in which humans are constructed as both different from and superior to non-human animals. Difference in this instance has been emphasized to the point that humans are frequently imagined to be outside of the animal category altogether.

This mode of understanding humans has historical ties to religion, particularly Christianity, as noted by historian Lynn White (1967) in his oft-cited article, ‘The Historical Roots of Our Ecological Crisis’. We will turn to a more detailed discussion of White’s contribution below, but for the moment, call attention to the fact that the term ‘animal’ itself reflects the human/nature dualism, in which all ‘animals’ are grouped together to distinguish the ‘human’ as the superior species. As Val Plumwood writes,

The concept of the human is itself very heavily normative. The notion of being fully or properly human is made to carry enormous positive weight, usually with little examination of the assumptions behind this, or the inferiorisation of the class of non-humans this involves. (1993: 26)

We will consider a possible linguistic shift to ameliorate this later in the article.

Non-human animals are largely absent from theories of religion specifically and society more generally. Nonetheless, as Aaron S. Gross argues, ‘the animal’ has served as an ever-present tool to demarcate ‘the human’ phenomenon of religion, which has been a focal point for the social sciences since their early development (2015: 95, 98). Non-human animals are, pardon the pun, the elephant in the room in social theory, a necessary foil to emphasize the specialness of humans and indeed the very concept of ‘humanness’ itself rests on a sharp distinction between humans and all other animals. In reference to Durkheim, Gross states,

By deﬁnition, for Durkheim, animals are incapable of classiﬁcatory/conceptual thought. There is thus no need to bother oneself by looking in the horse’s mouth – the gulf between human and animal has been determined in advance of any analysis. In one of the most constraining moves in his theorization of religion, this viewpoint suggests that animals (...) have no place in the study of religion (at least as themselves). If he appears to speak of animals a great deal, he insists that this is only because animals are so useful as receptacles of human categorization. (2015: 72)

The human/animal divide present in Durkheim’s work, which excludes non-human animals as social actors in ‘human’ society, lingers in contemporary sociological studies of religion.^2^ We propose that sociologists of religion take up the recognition of this implicit dependence on the human/animal divide, and its attendant hierarchies of life on earth, as they engage in a reinvigorated and critical sociology of religion such as that called for by Altglas and Wood (2018). In the process of engaging with other subfields and debates, sociologists of religion can contribute to a broader conversation about the implications of excluding non-human animals from our conceptualizations of the social. To be sure, we do not suggest that sociologists develop a new subfield for human/non-human animal relations. Instead, sociologists need to reconfigure *how we do sociology*, so that we no longer ignore or diminish the influence of other subjects in human social life.

## Non-human animals in sociology of religion

Animals make meaningful contributions to the lived experiences, practices, and narratives of those who identify as religious and those without any religious affiliation. Humans likewise impact the social lives of non-human animals, including their well-being and organizational structures.^3^ Concern for non-human animals sometimes prompts human beings, both religious and nonreligious, to collaborate across difference, generating new kinds of social and organizational structures that are driven by the realities of non-human animals (Beaman, 2017b). Yet, non-human animals are rarely mentioned in analyses of social life. They either appear as the core object of analysis or not at all. Altglas and Wood state that ‘sociology, as a discipline, will study religion as part of the social construction of the reality human beings live in. It is certainly not the only way to investigate the human world; this is, however, what the discipline of sociology implies’ (2018: 26). While we agree with this line of reasoning, we extend the notion of social construction to include non-human subjects. More precisely, we recognize that human beings live in a more-than-human world. Human social life is imbricated with that of non-humans. For the most part, sociology of religion and sociology, more generally, have treated human beings as though they live in a vacuum or in isolation from non-human beings. By excluding animals from social scientific inquiry, sociologists cannot adequately formulate a comprehensive understanding of how social life is shaped, experienced, and expressed. Such an impoverished approach has left us woefully unequipped to contribute to climate crisis responses, among other things, and has quite likely contributed to the hierarchical social imaginary that has caused the crisis in the first place.

We are not the first to note the absence of non-human animals in sociology. In 1979, Clifton D. Bryant wrote ‘The Zoological Connection: Animal-Related Human Behavior’, in which he argued that attention to the ‘zoological’ connection would open ‘new vistas of behavioral linkages and social causation’ (1979: 399). He illuminated the ways in which non-human animals saturate our language, our material culture, and influence our behavior. Indeed, his article is worth reading for its encyclopedic rehearsal of the diverse ways that this is so. Similarly, he discusses ‘the animal’ as social problem, as sentient being, as implicated in work, as surrogate human, and as implicated in crime. Perhaps presciently, he noted ‘animals are often a constituent indirect factor in human illness and in this connection, social epidemiologists have documented the epizootic relationship between man [sic] and beasts’ (Bryant, 1979: 400), an observation that seems especially relevant in these pandemic times. Bryant was critical of sociological myopia when it comes to non-human animals, noting their failure to take into account ‘the permeating social influence of animals in our larger cultural fabric’, and his arguments make a compelling case for sociological attention to non-human animals (1979: 400).

Bryant also devotes attention to the animal liberation movement that followed Singer’s (1975) *Animal Liberation*, remarking on how it challenges dominant cultural values related to non-human animals and the systems that keep them oppressed in captivity. This includes, for example, an increase in opposition to the confinement and use of animals in laboratory testing, which has become a point of concern for some research scientists. Bryant goes on to state: ‘Although sociologists have traditionally found social controversy of engaging scientific interest, they have apparently viewed animal related controversy, social movements, or collective behavior as possessing little sociological relevance’ (1979: 408). This issue has been addressed by more recent scholarship that studies animal welfare, animal liberation, animal rights, and (ethical) veganism as social movements in addition to individual identities or philosophies (Jasper and Nelkin, 1992; Johnston and Johnston, 2017; Munro, 2005, 2012; Wrenn, 2019a, 2019b). We are likewise concerned with the sociological relevance of animals, but we direct our attention away from broader social movements and toward the sphere of lived experience.

However, 30 years after Bryant’s work, another plea was issued for sociological attention to non-human animals. In their 2018 article ‘The animal challenge to sociology’, Bob Carter and Nickie Charles point to three reasons that sociology has failed to include non-human animals as a matter of course in social theory or analyses. These may help to clarify why other disciplines, such as anthropology, philosophy, and history, have been open to animals, while sociologists have largely ‘displayed a remarkable reluctance’ to expand the realm of social relations (Sanders and Arluke, 1993: 377). First, sociology has left responsibility for non-human animals to biology: ‘Animals, in particular, were defined as the concern of biology rather than sociology, despite their importance to the constitution of societies’.^4^ The second reason identified by Carter and Charles is assumptions about human exceptionalism – a position which had narrow ideas about who was to be included in the category of ‘the human’. Sociology’s highly problematic (our characterization, not Carter and Charles’) responses to Darwinism included Spencer’s Social Darwinism and theories of eugenics that were enthusiastically deployed against women and racialized groups, including Indigenous peoples, people with disabilities, ‘homosexuals’, Jews, and other marginalized peoples. Human exceptionalism in this context can be seen as not only informing an exclusionary definition of ‘the human’ but also as functioning to support the intimate linkages of hierarchies such as race, culture, sex, gender, *and* species.^5^

Carter and Charles argue that sociology’s emergence during accelerated industrialization and urbanization further alienated sociology from understanding the entangled lives of humans and non-human animals. Society for them cannot be adequately conceptualized without adjusting for this gap and that non-human animals ‘are agents entangled in relations with humans and that these relations are frequently ones of domination and exploitation’ (Carter and Charles, 2018: 80). Rather than entering the abyss of conceptualizing nature, Carter and Charles advocate for a reconceptualization of the social that includes non-human animals. Sociology is currently so far from such a reconceptualization that it is difficult to imagine how this might translate in relation to research in real terms. Sociological eyes glaze over at the mention of non-human animals, with the exception of a small group of scholars in sociology of animals, the environment, and related fields. There is a subfield of ‘human–animal studies’ that includes sociologists (Sanders, 2007), but as with many subfields, this remains a relatively marginalized and niche field, although there is some indication that this is changing (Wilkie, 2015). Non-human animals have not figured in any meaningful way in broader theoretical developments, with a few notable exceptions. We are not proposing yet another subfield, but rather an overhaul. For this, we draw on a number of scholars whose work is shaping a new direction in the scholarly imagination of what it means to be human in the world.

One such contribution is by Bruno Latour (sociologist and transdisciplinary scholar) who has reflected extensively on humans and the planet in his recent work. Latour’s project is to put humans back ‘in’ the world. It would be an understatement to describe his framework as profoundly relational. Latour’s work is not ‘the’ solution to the problem we identify. Rather, his reflections push us to ask the questions that need to be asked and offer some possible directions. For example, his proposal that we shift language to reflect the fact that we are all in ‘this’ together through the adoption of the word ‘terrestrials’, that is, that human and non-human animals constitute an indivisible category. Latour’s terminology displaces the human/non-human animal binary and forces us to consider everyone together, at once, and as inseparably linked in past, present, and future on the earth.

Political theorist William Connolly has taken a similar path in his reflections on a ‘world of becoming’. Connolly too seeks to disrupt boundaries in productive ways, critiquing the ‘constraints of species provincialism’ and identifying the possibility that: ‘At some times and for some purposes you explore how other modes of agency have served as precursors to human agency as they also enter into its layered character now’ (2011: 32).^6^ Connolly wishes to displace the ‘masterful human agent’ to open space for a much more nuanced and complex conceptualization of the social world which he understands to be a ‘world of becoming’.

The lacuna we identify is even more pronounced in sociology of religion. To be sure, there is a literature that examines human/nature relationships more broadly, some of it situated squarely in sociology of religion and some of a more anthropological nature.^7^ Non-human animals appear in this literature, but they are not central. Sarah MacFarland Taylor (2007) conducts ethnographic research with women of the Roman Catholic ‘green sisters’ movement in North America. She describes various efforts these sisters take to ‘harmonize with the earth’ through their daily practices. For two Medical Mission Sisters, this includes learning to accept the local deer who feed on produce from their organic garden (Taylor, 2007: 188). Taylor notes a rule at the Green Mountain Monastery which prohibits sisters from eating animal flesh unless they raised the animals themselves. The meat of animals who were ‘raised in cruel and unnatural conditions or who are forced to produce in ways that violate the integrity of their being’ are especially prohibited (Taylor, 2007: 165). Yet, the sisters largely understand their interactions with animals as one part of their larger harmonizing mission. Instead of being the focus of theological consideration, animals are subsumed into broader ideas of ‘the environment’, ‘nature’, or ‘creation’ more wholly.

Non-human animals now constitute an area of study in both theology and religious studies.^8^ It is well established that animals are integrated into the practice of religion (Waldau and Patton, 2006). They appear in customs and laws related to diet and slaughter (Dahlan-Taylor, 2015; Gross, 2012),^9^ stories such as those found in the Hebrew Bible (Stone, 2017) and Buddhist *Jātaka*s (Appleton, 2019), and rituals like pet blessings (Hobgood-Oster, 2008).^10^ From a more theoretical perspective, non-human animals first appear in literature concerned with ritual killing, particularly by René Girard (1977) and Walter Burkert (1983), who were interested in the origins and communal function of sacrifice. Ritual sacrifice later appears in scholarship addressing animal ethics (Waldau, 2002), an area that considers the human treatment of animals in both religious thought (Linzey and Linzey, 2019) and more concretely in practice and institutions (Govindrajan, 2018; Narayanan, 2018).

Although non-human animals are traditionally displaced as social actors in the sociology of religion, they are not entirely absent. Rattlesnakes, for example, play a prominent role in Robert Orsi’s (2005) ‘Snakes Alive’ chapter in *Between Heaven and Earth*. Orsi considers how Christians in southern Appalachia who handle snakes as part of their religious practice are portrayed in a preceding work. He argues that the author presents the snake handlers as ‘radical others’ whose religion falls on the inferior side of a moral boundary between ‘good’ and ‘bad’ religion. Those who practice ‘good religion’ are concerned with the mind and spirit, not bodily practices like gripping snakes while charged with religious ecstasy (Orsi, 2005: 188). Orsi explains that what counts as ‘good religion’ is steeped in the moral and political history of Protestantism. This history has shaped the study of religion to carry ‘unacknowledged assumptions, prejudices, and implications in power’ (Orsi, 2005: 180). These factors are perhaps most prominent when anthropologists developed the study of comparative religions during colonization. Orsi proposes an alternative approach he describes as postcolonial because it requires scholars to not ‘constitute the objects of our study as other’ (1997: 18). Instead, scholars of religion should conduct research ‘at the boundary between one’s own moral universe and the moral world of the other’ (Orsi, 2005: 198), which can only be accomplished by first exposing and abandoning the underlying moral biases of the discipline.

Orsi reframes the relationship between the snake-handling Christians and those who study them to challenge Western and Christian biases that so easily lead to their status as ‘others’. Yet, the boundary between the snakes as religious objects and their handlers as religious subjects evades critical examination. The snake-handling practice is described as dangerous due to the snakebites some practitioners may endure. However, the conditions that cause the snakes to bite in the first place are largely ignored. The snakes fall short of ethical consideration.^11^ What does it mean for the practice of the snake handlers to rely on the control of the snakes’ own bodies? What does it mean to think about a snake’s biting not only as dangerous but as an act of agency? Such questions rely on a conception of the snakes as subjects worthy of moral consideration. When reframing the snakes as subjects, we can also ask, how would this analysis change when the snakes are repositioned as an entry point? From a sociological perspective, the snakes can be conceptualized as social actors who organize human religious practice (albeit, presumably, unwillingly) around their bodies. They are not strictly ‘something dangerous’ being handled, but individuals who draw human social actors together. This includes those who belong to the church and outside visitors who arrive to watch the snakes being handled with fascination.

Although sociology of religion needs to reckon with the non-human animal gap on its own terms, we might also consider some of the literature from religious studies on this topic, both in terms of possible directions for sociology of religion and to identify less fruitful options. The latter includes imagining certain non-human animals as experiencing religion or a sense of spirituality. This approach places religion above social context by theorizing it as having an ‘essence’. Indeed, some religious studies scholars posit a ‘religion-like’ direction that expands the conceptualization of religion to incorporate nature and non-human animals.^12^ We are suggesting a different direction, inspired by the writing of Lynn White. Much of the literature on religion and ecology either implicitly or explicitly references Lynn White’s powerful and controversial essay, ‘The Historical Roots of Our Ecological Crisis’, first presented in 1966 and subsequently published in *Science* in 1967. Although he identified primarily as an historian, White specialized in social change and had a master’s degree from Union Theological Seminary. Religion was integral to his social analysis, including his work on technology. Like Weber, he explicitly linked Christian doctrine to social structure and social change. He also integrated natural science knowledge of his day into his work, which was both historical and concerned with present day crises, particularly that of the environment.

In his exploration of the ecological crisis, White suggested that it was necessary to look at the ‘presuppositions that underlie modern technology and science’. Positioning his observations in relation to technological developments, such as cross-plowing, White (1967: 1205) states, ‘What people do about their ecology depends on what they think about themselves in relation to things around them. Human ecology is deeply conditioned by beliefs about our nature and destiny, that is, by religion’ (1967: 1205). White argues that despite claims that we live in a post-Christian age (remember he is writing in 1967), we continue to live ‘very largely in a context of Christian axioms’ (1967: 1205). Changes in thinking and language ‘have largely ceased to be Christian, but to my eye the substance often remains amazingly akin to that of the past’ (White, 1967: 1205). And then comes the crux of his argument which continues to cause ripple effects and, as we have already noted, has yet to be addressed adequately by sociologists: ‘Both our present science and our present technology are so tinctured with orthodox Christian arrogance toward nature that no solution for our ecological crisis can be expected from them alone’ (White, 1967: 1207). White emphasizes the Christian posture toward non-human animals as one of dominance. While he writes broadly about the ecological crisis, he clearly sees the human/non-human animal relationship as part of this broader discussion. The two are inseparable.

Sociology of religion and indeed sociology more broadly replicate the shortcomings identified by White. We contend that the failure to incorporate non-human animals in sociological considerations of religion and nonreligion replicates a hierarchical model that sees human life as above or higher than non-human life. The exclusion of religion is a critical omission in the discussion by Carter and Charles. The entanglement of sociology with theories of exceptionalism was, to be sure, inspired by Darwin’s theories and the sociological fear that biology would usurp its authority (such as it was). However, it was not merely a matter of competing theories. The social sciences developed in Christian societies at a time of rapid colonization. Rationalizations for colonization were heavily dependent on a narrative of human exceptionalism that coupled being fully human with christianization (Cox, 2016). Denying human exceptionalism meant challenging the Christian foundation of society. There are two qualifiers to this point. First, within Christianity there are traditions, both ideological and practical, that soften the rigid hierarchies traditionally associated with Christianity. For instance, Pope (2015) encyclical on the ecological crisis criticizes the ‘excessive anthropocentrism’ of modernity and calls for a ‘sense of responsible stewardship’ (para. 116). Humans as stewards are better equipped to ‘protect nature’ and simultaneously tackle poverty caused by environmental degradation (para. 139). More practical translations of religious values into ecological action can be seen at the grassroots level with groups such as the Green Sisters, mentioned above, and organizations, such as A Rocha.^13^

The second qualifier or nuance is that sociology’s founding ‘fathers’ themselves had complicated relationships with that Christian foundation: Max Weber confessed to being ‘absolutely unmusical religiously’ and ‘neither antireligious *nor irreligious*’ (Weber, 2017: 324, emphasis original); Marx, of both Jewish and Christian origins, linked religion with capitalism (as did Weber) but negatively, arguing that it as a tool for maintaining social inequality; and Durkheim was an agnostic who saw the importance of religion in society, most especially as a key element of social cohesion. Although we cannot understand sociology solely in terms of these three thinkers, they have been extremely influential on the field. Ultimately, the embedding of Christian ideals of the world order, including the place of humans, infiltrates sociology. None of them considered non-human animals in everyday life, although Durkheim did if only to emphasize the differences between them.

We are not proposing to transform sociologists into biologists, but to reconsider the social as a broader field which would include, explicitly, human relationships with the more-than-human and a shared territory on which we engage in those relations. In his Levinas-inspired discussion of relational morality, Douglas Ezzy (2013) reports the initial awkwardness of asking the question ‘What Matters to Wombats?’ to his students as a way to introduce non-humans into the ethics that emerge from relationships. This awkwardness tells us a great deal about where we are in our conceptualization of the social. That we cannot ask about a non-human animal’s perspective without feeling weird or being made to feel weird by those around us, should trouble us and prompt critical reflection. It is important to identify this discomfort, to name it, and to consider its sources.^14^ We also acknowledge that this is a time of linguistic transition and so some level of awkwardness is to be expected as we search for words and phrases that better express a horizontal positionality.

Like Ezzy, Rosi Braidotti foregrounds relationships as the foundation for the emergence of ethics and highlights radial immanence and the ‘act of unfolding the self onto the world, while enfolding the world within’ (2013: 193). Resonances of Latour are present in Braidotti’s work, particularly in her emphasis on attachment and connection ‘to a shared world, a territorial space: urban, social, psychic, ecological, planetary as it may be’ (2013: 193). Braidotti couches her vision in the language of the posthuman, which she insists is not indifference to humans or dehumanization, but rather a ‘new way of combining ethical values with the well-being of an enlarged sense of community, which includes one’s territorial or environmental inter-connections’ (2013: 190). Braidotti, Latour, Connolly, and Ezzy are all advocating for an approach that emphasizes horizontal rather than vertical relations and that locates ethics, values, and morality in immanent rather than transcendent relationships (see also Bauman, 2005; Beaman, 2017a). Moreover, their ideas are linked to ‘hope’, something Braidotti explicitly champions. Rather than focusing on the negative, they emphasize the possibilities of collaboration and cooperation. The shift they champion is urgently needed for a robust response to the climate crisis which threatens not only human life, but, more importantly, the lives of everyone around us. As Carter and Charles conclude,

A fuller recognition of what humans and other animals have in common, that social life and culture are not uniquely human, is part of the animal challenge to sociology. And if sociology fails to respond to this challenge, it is our contention that it will be ill-equipped to address the pressing problems of the Anthropocene. (2018: 93)

There is precedent in sociology of religion for moving toward a horizontal conceptualization of the social that includes non-human beings. For example, in her book *Lived Religion*, Meredith B. McGuire focuses on everyday practice, opening space for us to (re)consider the place of non-humans in daily life. She details the spiritual importance of gardening for one of her participants:

Margaret, who called her daily work in the garden her ‘worship service’, described deeply spiritual connections she made when physically touching the earth (which is why, whenever possible, she gardens without gloves). Other senses were involved in her meditative practice, too, especially smell and sight – the smell of the air after a summer shower, the sight of a butterfly hovering over a flower, the sounds of frogs chirping from a nearby pond, the feel of the dirt-covered skins of newly dug potatoes, and the many shades of green. (McGuire, 2008: 110)

Margaret’s ‘spiritual connection’ is tied to her practice of gardening, which by definition requires close, purposeful interactions with plants. Air, soil, butterflies, and frogs are also contributors to her experience of gardening as meditative. McGuire does not focus her analysis on the role of terrestrials in shaping lived (religious) experience, but she does make clear that non-humans can be central to practices understood as spiritual or religious. This is a bit closer to the ‘religion-like’ direction from religious studies we have mentioned above, but it does open the possibility of theorizing from a more terrestrial-oriented stance.

Relationships between terrestrials come to the fore in more recent work in sociology of religion. Lori G. Beaman (2017b) explores how (non)religious sea turtle rescue volunteers frame their relationships to sea turtles and other non-human animals. Among other findings, Beaman notes that many rescuers used language of equality when talking about sea turtles, and they ‘combined science, emotion, and a sense of awe about the world in which they live’ (2017a: 21). This latter finding resonates with Anna Halafoff’s (2017) pondering of the awe humans may experience when encountering whales. She asks, ‘But what is it about whales that people, including myself equate with wonder, awe, bliss, and deep knowing? Is it that they are simply part of the natural world which inspires awe and wonder in most people?’ Emotional experiences like awe, or what Beaman (2021) theorizes as ‘enchantment’, can be understood from religious and nonreligious perspectives (De Groot and Van den Born, 2007; Thurfjell et al., 2019). Furthermore, the experiences one has with ‘wild’ sea turtles and whales may differ with those who are ‘captive’ at aquariums. As we begin to take human/non-human relations more seriously, paying attention to context is important to avoid essentializing perspectives of and encounters with other animals, whether these are exceptional or mundane.

As we sort through the messy entanglements of social life at multiple analytical levels (individual, group, institutional), we ask how human relations with non-human beings are imbricated in these analytical fora. This includes how people make sense of and respond to their position vis-à-vis other terrestrials. An example of research that draws on this sensibility is Timothy Stacey’s (2021) project with the Metro Vancouver Alliance. Stacey examines practices that are neither obviously religious or nonreligious, and in the process showcases ‘tools from the study of religion for the broader social scientific study of world repairing work’ (2021: 91). His interlocuters frame their actions as being about restoring the dignity of people and the planet which are, in their understanding, intertwined. Stacey captures the collaborative core of the shared work of the people he interviews (and spends time with) which is recognized by them as taking place on Indigenous territory, and in a resource-extractive capitalist context that ‘depletes the waterways and disembowels the land that Indigenous people hold sacred’ (2021: 94). Stacey’s research captures what is at the heart of sociology of religion in many ways: studying what is important to people by studying social action. A comprehensive understanding of this action requires a consideration of the place or status of the non-human in its composition.

## Recrafting the focus

Incorporating non-human animals into sociology of religion requires a reformulation of the field’s underlying anthropocentric structure. This call comes at a time when space for reformulation is already being made by sociologists turning their attention toward nonreligion. The rise in those who identify as having no religion has challenged sociologists to rethink how they study human experiences, beliefs, practices, relationships, and morals. In particular, complex articulations of morality by the nonreligious pose a challenge to the sentiment that morality is tied to religion. As Ezzy explains, ‘The way we study [religion and nonreligion] can easily mask privilege, and in particular Christian privilege, through the historical influence of Christian assumptions about the academic study of religion. These include the assumption that nonreligious people are not moral’ (2021: 149). An implicit hierarchy exists between the religious and those ‘lacking religion’ and its moral framework. As sociologists seek to better understand the nonreligious, including their moral frameworks and relationships, they might also correct the sentiment that agency, subjecthood, and social relations are unique to human beings.

We want to conclude our discussion with a modest contribution to the conceptualization of a terrestrial approach. We draw on our research experience in the Nonreligion in a Complex Future project. One of the five core areas of investigation on that project is ‘the environment’. Our explorations on this topic include, thus far, two projects. The first, Experiencing Nature During Physical Activities, includes a survey of walking and hiking practices in our participant countries.^15^ Several of our survey questions are designed to capture more qualitative data, including a question that asks participants to describe a memorable experience they had in nature. These memories offer us insights into how individuals conceptualize nature and their relationship to it. Some of our participants recalled encounters with non-human animals. For instance, one participant wrote,

An encounter with a black rat snake stands out. She was threatened by us initially, rattling her tale in the leaves to mimic a rattlesnake. When we quietly sat down and didn’t make any aggressive display she gradually calmed down and then proceeded to go about her business which was to climb a tree near us. We got to watch her negotiate the various branches and when she got up to head height we stood up to take her picture and she curiously extended her head and neck towards us. It was a very special moment of what felt like a true connection.

The snake is at the center of this story, a relational partner in the encounter as ‘she’ (not ‘it’) who makes decisions and engages with the humans on her own terms (‘she curiously extended her head and neck toward us’). This chance encounter resulted in a ‘connection’ between terrestrials, as opposed to a human merely witnessing an animal. The story as told demonstrates a horizontal conceptualization of the snake and her world: we see traces of humility, deconstruction of a human/animal binary, and respect for the snake as a fellow terrestrial.

Our second example is from the Community Gardens project. In this project, we are interested in both human-to-human relationships and cross-species encounters that take place at community gardens. Almost all our gardeners mention non-human animals as companions, pests, sources of awe and wonder, and so on. One interviewee described how he works to establish a reciprocal relationship in his community garden. This entails giving back to the non-human animals who live there, such as by leaving weeds to grow which other animals or insects find valuable. Difficulty arises when other members do not share their outlook. In one instance, some potato bugs were found on a couple of potato plants, and some volunteers responded by squishing them all. The interviewee described this as a bit of a conflict because he would have preferred ‘to let things run their course and find balance on their own over time’. Another participant, when describing how raccoons ate the corn at her community garden prior to harvest, stated,

The fact that the raccoons came in and ate the corn, it was a shame. But I think there’s a sense that we share the park and the environment with other living things. Certainly, there are snakes there and birds there (...) Even though you try to put up netting or you try to ward them off you can’t. It just still happens. You take it with a grain of salt.

Potato bugs, raccoons, snakes, birds, and plants all appear as actors who shape the experiences of the humans we interview. They are not only present at the garden but help form the terrestrial relationships that constitute social life at the garden.

Articulating the importance of the other-than-human in analyzing our results is only possibly at a cursory level here but suffice it to say that shifting our point of entry, from the human to the other-than-human, allows us to think about life differently. For example, as we began the process of coding our interviews, we thought about how our participants’ lives intertwined with the plants they grow. For the Syrian refugees in one garden, this was often a direct link to ‘home’. Some had been farmers in their lives in Syria, and the small garden plots they tended in Canada were a micro version of those much larger gardens. The community garden also provided a window into that other life and a continuity with it as well. It allowed them to reconstruct home. For an herbalist we spoke to, some plants were cuttings from her mother’s garden. For other gardeners, the plants they grew evoked memories of friends, family, and experiences in Syria. Furthermore, sharing seeds and plants linked the people in these communal spaces – the plants acted as their shared language, when sometimes it was impossible to speak to each other. None of these relations would have existed without the plants: without the plant there is no connection, no memory, and no reconstituted relationship. The plant becomes a necessary part of the story, a conveyer of meaning, and a key actor in the narrative.

By writing non-human beings out of the social, social scientists support a narrative that valorizes the human, missing key relationships and catalysts of meaning and action. By recognizing the centrality of non-human animals and plants or, to use Latour’s notion, by reshaping analysis to begin with terrestrials, sociology participates in and contributes to the shift necessary for the difficult times ahead as we face the realities of the climate crisis. In response to our invitation to write a brief ‘letter to nature’ in the Experiencing Nature During Physical Activities project, many participants used words like ‘respect’, ‘interconnected’, and ‘gratitude’:

I don’t believe I am separate from ‘nature’ but my feeling to express is gratitude, for the air in my lungs, the ground holding me up, the incredible web of life that surrounds me and I am a part of. Being in natural spaces revives and refreshes me and puts my life and stresses in perspective.

It is admittedly not terribly difficult to take the terrestrial route on the two research projects we have briefly mentioned given their subject matter. However, we began with the assumption that relationships across species groups shape individual, group, and institutional life. World-repairing work between religious and nonreligious humans is often shaped around the other-than-human and this will and must increasingly be the case due to climate change. All too often sociologists of religion have been in lock step with the religious groups they study in relation to hierarchical and anthropocentric conceptualizations of the world around us. Breaking with that conceptualization will allow us to see both the human and non-human elements of the social world.

## Conclusion

For the terrestrial approach we have in mind, we also draw inspiration from anthropologist Anna Tsing’s research drawing on the matsutake mushroom (or, as she says, ‘thinking with mushrooms’) as her entry point to exploring a world of social, economic, and political relations which includes the other-than-human (2015: 38). Tsing points out that the voices of other living beings are silent (i.e. we do not imagine them speaking) – ‘we imagine well-being without them. We trample over them for our advancement; we forget that collaborative survival requires cross-species coordinations’ (2015: 155–156). Like Latour and Connolly, Tsing focuses on the networks of relationships.

We have entitled this article ‘Toward Equality’ to reflect what we see as an emerging discourse in our empirical research, one which integrates a horizontal field of interaction and action that flattens the pervasive hierarchical framework within which humans and the world around them live. The shape of this equality is emerging, but begins, with the assumption that non-human animals (and in some cases, this is extended to plants and other ‘natural beings’, such as rivers and mountains) have an equal right to exist on this planet. Scientific attention to other-than-human animal sentience has accelerated in recent years, raising further questions, such as the relative weight of human and non-human animal life which has often been hierarchicalized on the basis of a lack of sentience in non-human animals. Similarly, research by scientists like Suzanne Simard (2021) has challenged our basic understanding of trees and their within and inter-species communication, raising questions about plant agency. Philosophers such as Sue Donaldson and Will Kymlicka (2011) have proposed a recalibration of rights based on non-human equality. As their work demonstrates, this shift necessitates a consideration of our fellow terrestrials in our concepts, theories, and research design.

We are not arguing for a mushroom at the heart of every story, or for a cat in every theory.^16^ Instead, we are calling for a more robust notion of situatedness and standpoint that will inevitably draw in, and sometimes begin with, the other-than-human. Likewise, we are not proposing that sociologists of religion focus on human/non-human animal relations as a distinct area of research. Proponents of animal rights have long called for equal moral consideration of animals. We are calling for equal consideration of animals as relational beings and accordingly social actors. Understanding the relationships at the core of human life, including those with non-human terrestrials, is central to understanding religion and nonreligion. A key step is to disassociate ourselves from the hierarchical arrangements of the religions we study and to eschew human exceptionalism. Humans are a species whose lives are entangled with the non-human world. We are one species among many. We end with the words of Carter and Charles (2018: 81), and hope that perhaps sociology is at last ready for the change they and we argue for:

[S]ociologists need to reconceptualize ‘society’, revise notions of agency, subjectivity and reflexivity, and reject the speciesism and anthropocentrism on which sociology is based. Finally, we contend that continuing to direct the sociological gaze only at humans significantly limits the sociological imagination and is in danger of rendering it irrelevant in the age of the Anthropocene.

## Notes

1 For this argument, we turn to the work of Carter and Charles (2018) below.

2 Due in part to its early subject focus on cultures that did not have the Western human/nature dichotomy, cultural or social anthropology offered alternative visions of human/non-human animal relationships. Philippe Descola’s (1996) *In the Society of Nature* marked a significant shift in complicating this dichotomy. Earlier works like that of Claude Levi-Strauss (1963) included animals to the extent that they offered insights into human social formations. For a concise overview of animals and social anthropology, see Molly Mullin (2002).

3 Non-human animal social lives have been researched by biologists. For examples, see Hal Whitehead (2008) and Muller and Harris (2021).

4 The seemingly endless ‘nature versus nurture’ debate surrounding ‘sociobiology’ (see Wilson, 1975) may also partially account for the sociological avoidance of non-human animals in its accounting of social life.

5 For scholarship addressing intersectional and interrelated hierarchies, see Carol J. Adams (1990), A. Breeze Harper (2010), Maneesha Deckha (2012, 2020), and Bénédicte Boisseron (2018).

6 This is one of the two prongs of approach to agency, the other being a focus on ‘how multiple fields with different degrees of agency help to constitute the larger world of becoming in which we are set’ (Connolly, 2011: 32).

7 In this article, we are concerned with Anglophone sociology. French sociologists have shown a much stronger interest in non-human animals from epistemological and empirical perspectives. See, for example, Rémy Chauvin (1982, 1984), Dominique Guillo (2006, 2009, 2015), and Emmanuel Gouabault and Claudine Burton-Jeangros (2010). See Jérôme Michalon et al. (2016) for an insightful article on how non-human animals appear in French sociology and their proposal of a ‘sociology with animals’.

8 Some of these works recognize the agency or subjecthood of animals within certain areas of religious traditions, but they still carry the conceptual baggage of the human/animal divide that excludes animals from serious or mainstream theoretical consideration. Exceptions include Gross’ definition of religion (2015: 96–97) as ‘a dimension of reality that might be seen, for example, not only in relations among members of the species *homo sapiens*, but in relationships *homo sapiens* have with non-human (and non-divine) beings, and in relationships among non-human species themselves’.

9 Mary Douglas (1993) offers an interpretation of the symbolic value of ‘forbidden animals’ and their classifications in relation to dietary laws found in the book of Leviticus.

10 See Perry and Burge (2020) for a study on Christianity and predictions of pet ownership in the United States.

11 Considering the ethical implications of the snake-handling described by Orsi is not to further the ‘othering’ of these Christians. It rather points toward the distance between non-human animals and scholars of religion.

12 Aaron S. Gross (2015) and Donovan O. Schaefer (2015) both theorize religion to include non-human animals. Others characterize human relations with nature (including non-human animals) as ‘religious’ or ‘spiritual’, even when they are not articulated as such by individuals. Bron Taylor takes this approach in *Dark Green Religion*, for which he employs a ‘family resemblance’ framework to label what he considers to be ‘religion-resembling phenomenon’ as ‘religion’ (Taylor, 2010). Taylor’s work is part of a line of scholarship, most recently articulated by Paul Bramadat (2022) and his concept of ‘reverential naturalism’, that imposes ‘religion-like’ (or more elusively ‘spiritual’) frames of meaning on human/non-human nature experiences.

13 A Rocha is an international Christian organization that leads community-based programs for environmental education and sustainable development, ranging from marine conservation to church-run community gardens.

14 Discomfort is an important theme of Marjorie Spiegel’s (1988) provocative and seminal work *The Dreaded Comparison*, which demonstrates similarities between rationalizations for the enslavement of Black people and the violent exploitation of non-human animals.

15 For more information, see https://nonreligionproject.ca/the-project/.

16 In reflecting on lockdown during the Covid-19 pandemic, Latour talks about the worlds to which we had access from the confines of our living spaces, including ‘the stories, courses, online purchases, and *felines*, namely cats, that came from this other world but that we couldn’t touch, feel or embrace’ (2018: 30). We acknowledge here Atún, an Argentina-based feline who is a regular participant in our Zoom project meetings.
